# What is different about spinal pain?

**DOI:** 10.1186/2045-709X-20-22

**Published:** 2012-07-05

**Authors:** Howard Vernon

**Affiliations:** 1 Canadian Memorial Chiropractic College, 6100 Leslie Street, Toronto, ON, M2H 3J1, Canada

**Keywords:** Pain, Spine, Peripheral limb, Somatotopy, Referred pain, Chronicity

## Abstract

**Background:**

The mechanisms subserving deep spinal pain have not been studied as well as those related to the skin and to deep pain in peripheral limb structures. The clinical phenomenology of deep spinal pain presents unique features which call for investigations which can explain these at a mechanistic level.

**Methods:**

Targeted searches of the literature were conducted and the relevant materials reviewed for applicability to the thesis that deep spinal pain is distinctive from deep pain in the peripheral limb structures. Topics related to the neuroanatomy and neurophysiology of deep spinal pain were organized in a hierarchical format for content review.

**Results:**

Since the 1980’s the innervation characteristics of the spinal joints and deep muscles have been elucidated. Afferent connections subserving pain have been identified in a distinctive somatotopic organization within the spinal cord whereby afferents from deep spinal tissues terminate primarily in the lateral dorsal horn while those from deep peripheral tissues terminate primarily in the medial dorsal horn. Mechanisms underlying the clinical phenomena of referred pain from the spine, poor localization of spinal pain and chronicity of spine pain have emerged from the literature and are reviewed here, especially emphasizing the somatotopic organization and hyperconvergence of dorsal horn “low back (spinal) neurons”. Taken together, these findings provide preliminary support for the hypothesis that deep spine pain is different from deep pain arising from peripheral limb structures.

**Conclusions:**

This thesis addressed the question “what is different about spine pain?” Neuroanatomic and neurophysiologic findings from studies in the last twenty years provide preliminary support for the thesis that deep spine pain is different from deep pain arising from peripheral limb structures.

## Introduction

### Case scenario

A 45-year old male presents with chronic lumbosacral pain and some pain in the posterior thigh. Examination rules out any overt disc herniation with radiculopathy. X-rays show no spinal pathology.

Basic differential diagnosis options:

1. Back pain with referred leg pain (one problem with two clinical manifestations: one primary, the other secondary)

2. Back pain and an associated, but not causally connected problem in the posterior thigh (two separate problems)

Both of these options share an acceptable clinical logic; their distinction would be made on the basis of further history and examination for signs that were consistent with one or the other explanation.

However, there is a third option to consider:

3. Primary problem in the thigh referring pain to the back (reverse circumstances to #1).

It is my contention that this third option does not enjoy the same “clinical logicalness” as the first two, and would very likely not even be entertained as a possibility.

In this paper, I would like to explore why this is so. The answer that compels itself is rather simple, but not widely accepted to date: pain from deep spinal tissues is different than pain from deep tissues of the peripheral somatic structures (upper and lower limbs as well as the facial region), and the nature of referred pain from these structures is one of the critical distinctions.

### Background

In the early 1990’s Gillette et al. [1-8] began an exploration of dorsal horn neurons responsive to afferent inputs from the deep tissues of and around the spinal column. They referred to these tissues as “axial” in origin; they included intervertebral disc, facet joint capsule, deep intersegmental muscles, spinal dura (including the nerve root sheath) and sympathetic trunk and other nerve structures. Their work was motivated by the observation of what they described as a distinctive “clinical phenomenology” of spinal pain. The features of this clinical phenomenology are shown in Table 1.

**Table 1:** Clinical attributes of somatic (non-radicular) spinal pain

**Symptoms**	**Attributes**
• Deep, dull pain often spreading	• Chronicity
• Often bilateral	• Poorer discriminability
• Pain referral is common and can often involve large zones of referral to distal tissues	• Recurrence

The primary mechanism identified by Gillette et al. [8] to explain the distinctive features of spinal pain was the phenomenon of multiple convergent afferent terminations on subsets of dorsal horn neurons. They termed these subsets of neurons “low back neurons” and they identified numerous sources of convergent afferent input onto these neurons from superficial cutaneous sources, deep axial somatic sources (from the tissues identified above), deep distal somatic structures (muscles and joints of the lower limb), visceral structures, spinal dura and from the sympathetic fibres in the lumbar area. Gillette et al. characterized these low back neurons as “hyperconvergent”; thus, explaining the diffuse spread and poor localization of spinal pain as well as the centrifugal (distally-directed) tendency of somatic referral of spinal pain to multiple distal sources (depending on the spinal level of origin).

Since this work was published, much more has come to be known about the neuroanatomy and neurophysiology of spinal pain. A much more detailed, but enriched explanation can now be presented for the clinical phenomenology first distinguished by Gillette et al. This paper will present a synopsis of this work toward elucidating the thesis that ***deep pain of spinal origin is different from deep somatic pain arising from the peripheral limbs***.

The attributes listed in Table 1 also share many features with deep somatic pain in general. Indeed, in 2003, a leading authority on muscular pain, S. Mense, authored a paper entitled, “What is different about muscle pain?” [9]. Mense summarized the growing body of work from the mid-1980’s on deep somatic / muscular pain which clearly distinguished it from superficial, cutaneous pain, particularly on its quality (deep, dull aching) and localization (poorer discriminability). Since spinal somatic pain arises from the same types of deep tissues as discussed by Mense, it is natural that spinal pain would share those features as well. In fact, it is a corollary of my primary thesis that pain specialists and neuroscientists have tended to regard spinal pain as just another example of the “deep somatic pain” category, with no other distinctive features. In the past, it appears to have been sufficient to acknowledge that spinal tissues have nociceptor innervations [10-21], and then apply the now-standard understanding of deep muscle and joint pain. In this regard, no special attributes of pain from spinal/axial tissues were considered.

In fact, in a slightly earlier article, Mense had attempted to present an argument for why low back pain often becomes chronic [22]. Interestingly, the entire argument was based on the distinctive features of deep somatic pain in general which might explain chronicity (citing many of the features that were subsequently included in the 2003 article); there was no reference to a single distinctive feature of pain from spinal deep tissues. The argument was basically that back pain is problematic and can become chronic because deep muscular pain is the problem. Since the spine has deep muscular structures, spinal pain is like pain from any of the other deep somatic tissues of the musculoskeletal system.

In order to elucidate my thesis, I will advance another sub-thesis. If there really are any differences between pain of deep spinal origin vs deep peripheral pain, these differences must be based upon empirically demonstrable differences in the neuroanatomy of spinal pain and in the mechanisms of pain processing that subserve spinal pain. Furthermore, there should be enough important differences in these substrates to justify the assertion of a qualitative difference between spinal vs peripheral deep somatic pain.

Before starting the exposition on this thesis, some terminology needs to be defined. The association between a spinal region and one of the limb pairs exists classically for the cervical spine / upper limb and lumbar spine / lower limb. In these regions, three distinct sectors can be distinguished. The spinal sector is regarded as “axial”. The upper portion of each limb is termed “proximal” as compared to the lower portion being termed “distal”. These relationships are realized as: neck (cervical)/ shoulder/ forearm/hand and low back (lumbar)/ hip/ leg and foot. I propose that, based on embryology and neuroanatomy, a similar relationship can be posited for the upper cervical spine (axial)/ TMJoints (proximal)/ orofacial area. This latter proposition will be justified in due course.

The primary thesis of this paper can now be clarified as “deep somatic axial pain is different from proximal and distal limb deep somatic pain”.

## Discussion

### Clinical phenomenology of deep somatic spinal pain

I will first re-examine the clinical phenomenology of deep somatic spinal pain by concentrating on the issues of localizability, pain referral (especially with respect to extent and locations of pain referral) and chronicity. I acknowledge that, with respect to what have traditionally been regarded as “pain qualities” (deep, dull, aching characteristics vs sharp, burning characteristics), deep somatic spine pain is generally similar to any other source of deep somatic pain.

The questions asked here are: How is deep somatic spinal pain typically experienced by people with respect to its “where?” and its “with what?” and, “Why does spinal pain so frequently refer to distal sites and why does it so frequently persist and become chronic?”

1) Localization:

It is well established, with respect to another sensory modality – tactile sensation – that discriminability (as measured by 2-point discrimination) is much better in the distal vs the proximal portion of the limb [23]. An examination of the few studies which have also measured 2-point discrimination in the skin over the spine [24] shows even poorer precision in the relevant areas (neck vs arm, back vs leg). The most well-accepted explanations for the limb gradient in tactile discriminability are density of peripheral mechano-receptors (peripheral mechanism) and extent of representation of the region in the somato-sensory cortex (central mechanism). Does this gradient exist for pain as well? It would appear so, at least with regard to cutaneous pain, as Light and See (24) make the following statement:

"“The distortions in this map (somatotopic representation of body in spinal cord) also represent the density of primary afferent innervations of the skin, with digits being much more densely innervated than proximal limb regions and trunk regions. These distortions are correlated to nociceptive discrimination of these same body regions, with greater discrimination possible at the tips of the digits and less on the trunk”."

The degree to which this also applies to deep pain of spinal origin has, to the author’s knowledge, not been explored; however, it is likely to be the case. This would certainly be an area of fruitful research.

#### Referral of pain

Here, a crucial distinction needs to be made between pain radiating from the spine as a result of neuropathic pain arising from nerve root inflammation (radiculitis, radiculopathy) and pain arising from the deep spinal tissues, especially the deep ligaments, facet joints and deep intersegmental muscles, which is referred to sites distal from the spine. The mechanisms of pain radiating into the limbs resulting from inflammation of the nerve root sheath as a result of disc herniation or lateral entrapment are reasonably well-known, and are encompassed under the category of “neuropathic pain”. The nerve root sheath accompanies the mixed spinal nerve into and up to the lateral margin of the intervertebral foramen. This is the only site along any of the spinal nerves where a dural sleeve exists whose inflammation or irritation may result in pain which radiates along the dermatome of the affected nerve. My thesis does not pertain to this phenomenon. Rather, the concern here is with what Cramer has termed “somatic referred pain” [25], resulting from nociception arising in deep somatic tissues, especially the facet joints, the deep posterior disc structures and the deep intersegmental muscles.

As well, the thesis does not pertain to two other circumstances that involve what appears to be “referral” of pain. The first is any peripheral neural entrapment syndromes such as carpal tunnel syndrome or any other peripheral neuropathy. Again, these syndromes involve neurogenic pain which is to be distinguished from somatic pain, the focus of our thesis.

The second additional issue is that of clinical syndromes involving central pain mechanisms such as post-stroke pain or fibromyalgia. These issues will be taken up in the last section on “Challenges to thesis”.

**Figure 1:**
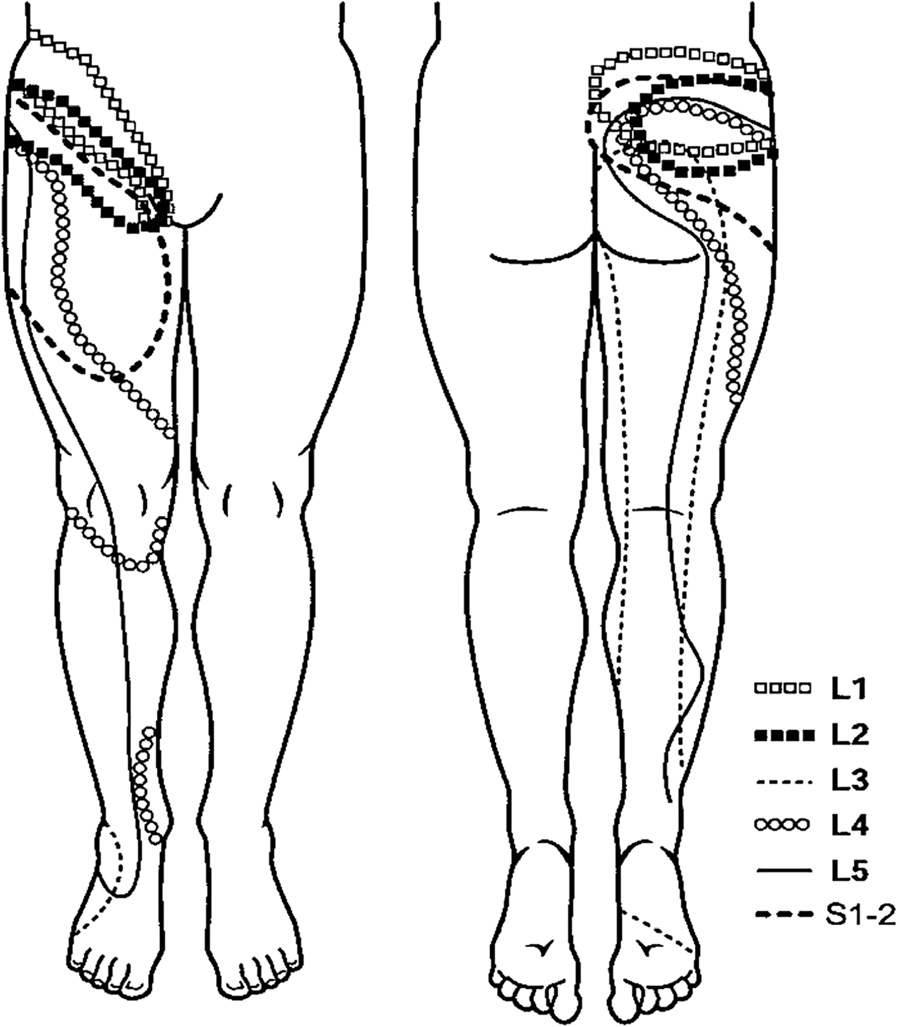
Zones of Head, in: Jinkins, 2004.

The history of the phenomenon of somatic referred pain [26-28] will not be reviewed here. Jinkins has reviewed the “zones of Head” in his 2004 paper [29,30], as shown in Figure 1. He has made the comparison between “Head’s Zones”, which classically mapped pain referral from visceral structures to the pain referral patterns resulting from deep spinal pain. The central feature shared by both categories of deep structures is that pain is referred in the sclerotome, much as Cramer has described the matter [25].

Several features of these somatic referral zones are important for the thesis. First, these referral zones result from deep somatic pain from the spinal tissues at each level. This point is crucial because, later in the thesis, pain arising from tissues in the peripheral limbs which may be innervated by one or another of the same neuromeric levels, but which do not present with the same pain phenomenology will be encountered. Here, the question asked is “how can pain input from spinal structures into the neuromere produce such different pain phenomenology from pain input from other structures into the same neuromere?” or, “why doesn’t pain from both kinds of structures produce the same pain phenomenology?”

**Figure 2:**
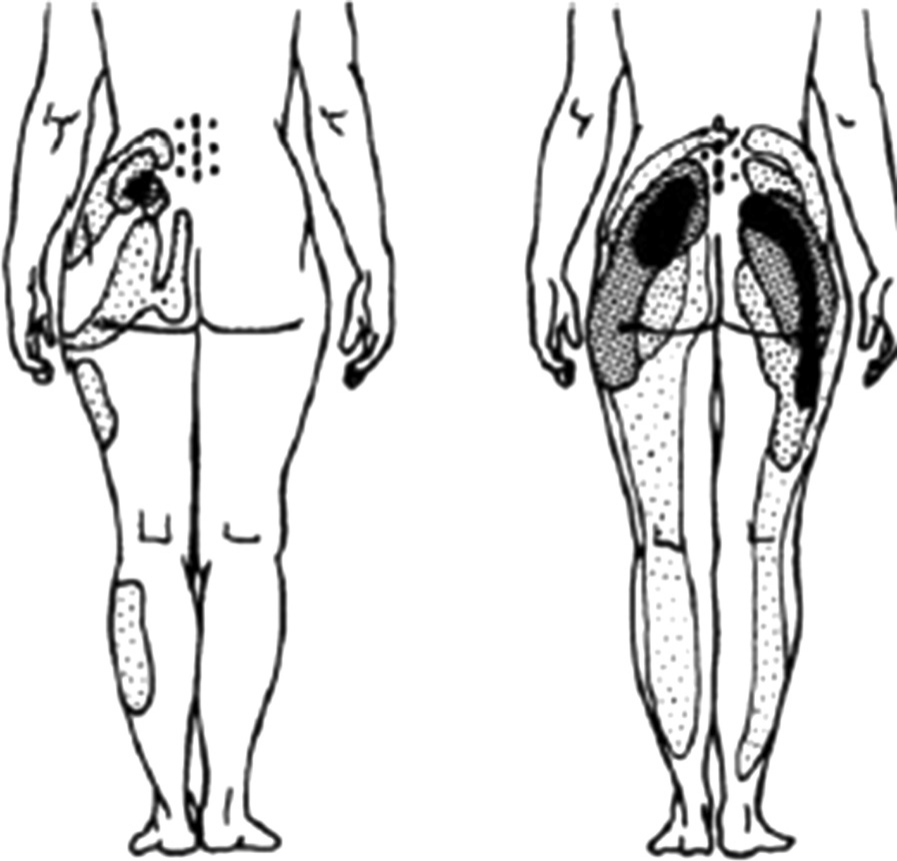
**Facet referral patterns of Mooney and Robertson [**[31]**].**

**Figure 3:**
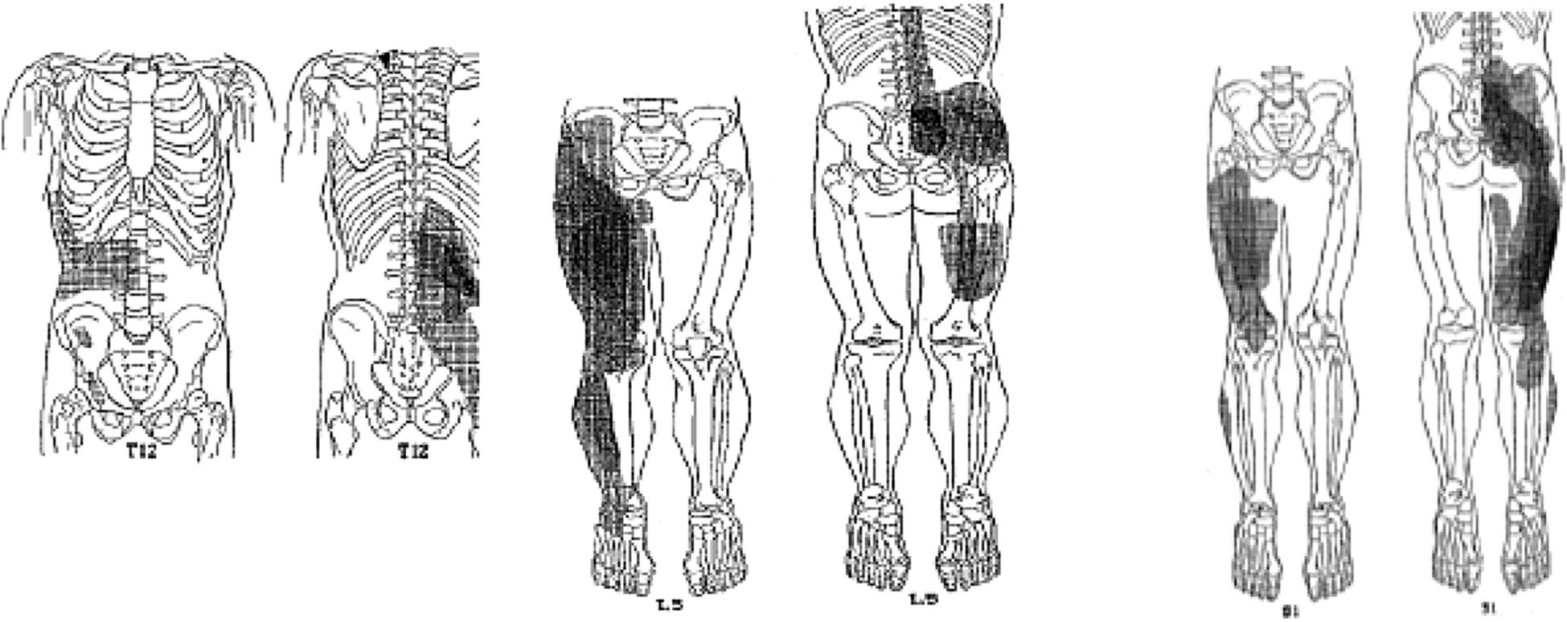
Pain referral zones from Feinstein, 1978. T12, L5, S1.

Second, the referral zones do not precisely match the neuromeric dermatome. In fact, they represent what is referred to as the “sclerotome” (See Figure 2, [31]). Third, referred pain extends into the anterior hip region from L1, 2 & 3 levels while all levels refer pain to the gluteal or posterior hip region. Fourth, referred pain from the L4, L5 & S1 segments extends beyond the knee. These levels also provide innervations to the sacroiliac joints, so these joints become important sources of “low back” pain referral. More precise pain referral zones from particular tissues have been identified by studies of algogenic irritation in normal and symptomatic subjects. Seminal work in the area of spinal facet joint pain was conducted by Feinstein [32]. Depictions of facet joint pain referral zones from Feinstein’s work are shown in Figure 3.

**Figure 4:**
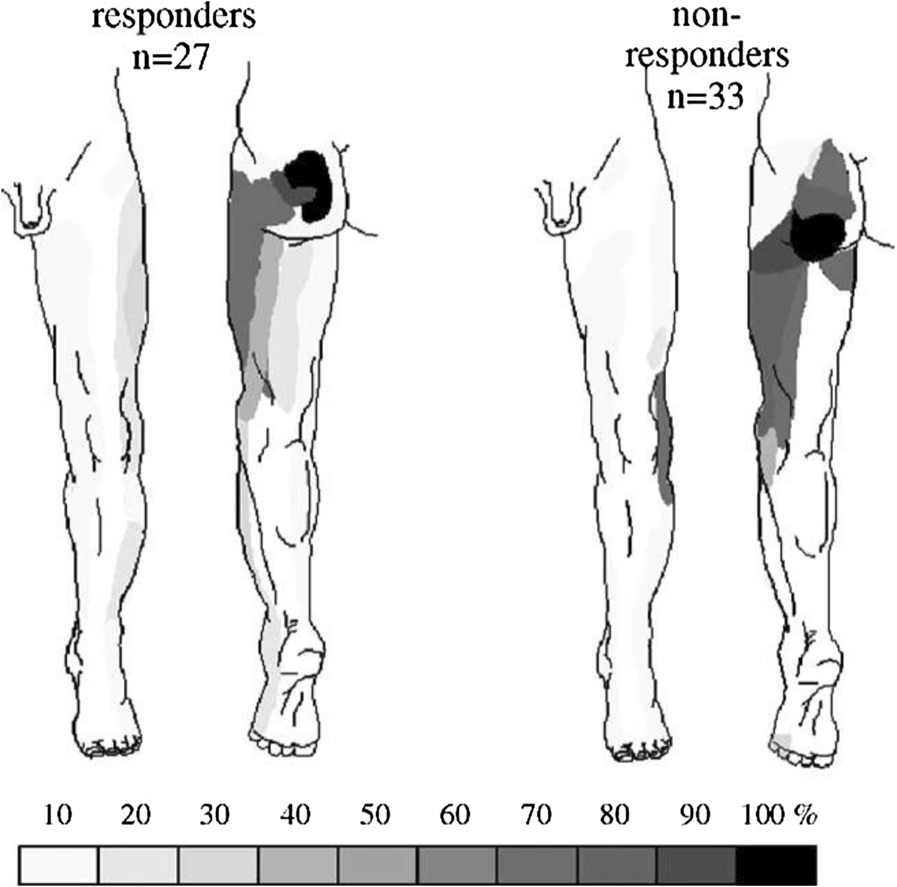
**Pain referral from the sacroiliac joint.** Van der Wurff et al., 2006 [33].

For sacroiliac joint pain referral, note the zones of anterior and posterior hip pain as well as the extension of pain past the knee to the ankle (See: Figure 4[33]).

The question of how often a local spinal pain generator in the deep somatic tissues produces a clinically demonstrable referral of pain is difficult to answer. Not all spinal pain complaints include pain referral, even if the experimental pain referral patterns are consistently evoked in test subjects. As well, some spinal pain conditions can be experienced as a sharper, more well-localized pain. This is especially so with costo-vertebral pain, although there may also be pain referral in these conditions as well (T4 syndrome [34]). It is generally accepted by clinicians that pain referral is common in spine pain conditions. As well, I will assert later that it is much more common than from the peripheral joints of the distal limb.

2) Chronicity:

An initial episode of spinal pain can, in many cases, persist and become chronic and/or recurrent. The questions of how often this actually occurs, and whether spinal pain becomes chronic to a larger degree than does pain from peripheral joints are difficult to answer. Recent studies of specific spinal pain complaints such as neck pain or low back pain indicate that spinal pain persistence at one year is much more common than was previously thought [35]. The recent report of the Neck Pain Task Force [36] characterizes neck pain (and by analogy, spinal pain in general) as a relapsing and remitting problem that persists well into a person’s life, with episodes of varying intensity and impact on daily life throughout one’s life.

The currently widely-accepted approach to the issue of chronicity in spinal pain is based on the “Biopsychosocial Model” [37,38], which places greatest emphasis on psychological issues in the development of chronicity. Indeed, many studies have found that certain psychological variables such as depression and catastrophizing strongly predict which subjects will have longer-lasting pain. Many so-called physical variables, such as pain intensity or x-ray findings do not predict chronicity anywhere near as strongly.

Much more recently, a new paradigm has emerged which re-focuses attention on what might be called biological features of spinal pain – namely, the model of central sensitization. This has been applied with great success by Sterling et al. [39-43] and others [44] to the problem of whiplash injury in the cervico-thoracic spine. This model, which is based on the theory that persistent pain induces sensitization of central pain processing pathways at the level of the spinal cord and above, lends itself much better to the current thesis, because it permits us to ask whether pain from spinal tissues induces central sensitization to any different degree than pain from peripheral joint tissues. This question will be addressed later in the paper.

### Peripheral joint pain phenomenology

**Figure 5:**
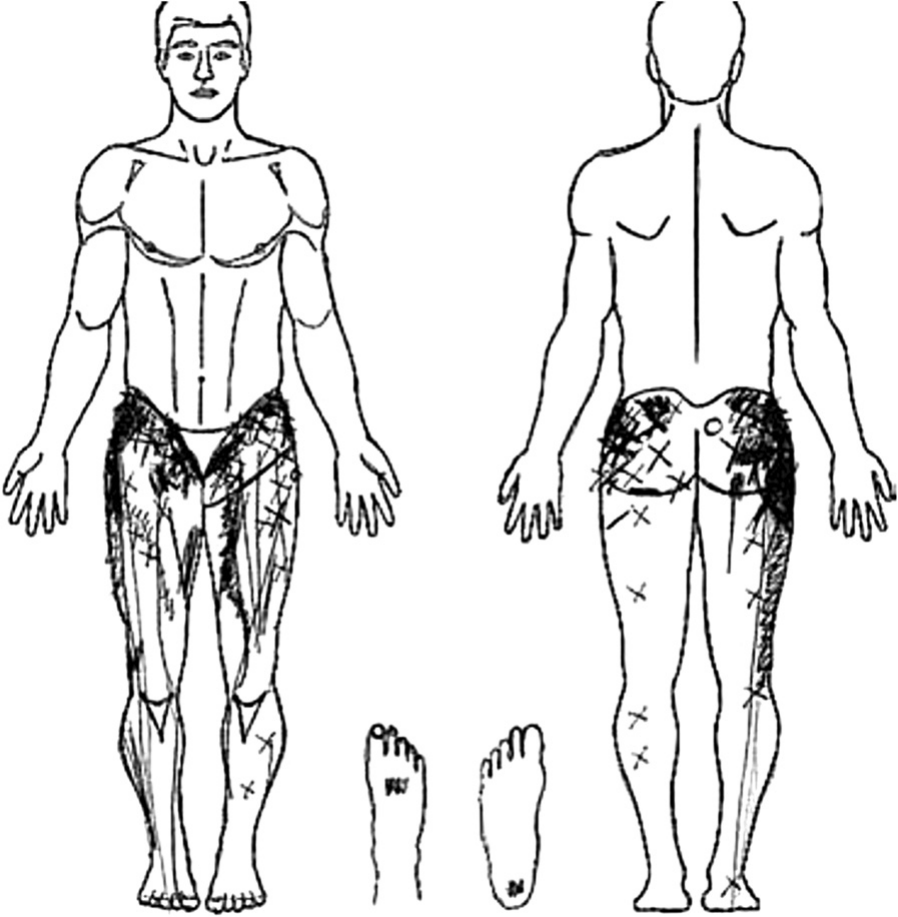
Hip pain referral: Lesher et al. Pain Med, 2008 (irritant injectant).

Here, we apply the categories of ‘proximal’ and ‘distal’ to the structures in the peripheral limbs. Hip pain is an example of a proximal joint. Lesher et al. [45] are amongst the few groups who have studied pain referral patterns from the hip joint. Importantly, these data were obtained from patients with hip pathology and clinical complaints of hip pain. Their depiction of their data is shown in Figure 5.

While the distribution of referred pain appears extensive, it is important to note that there is no referral of pain to the spine (in this case, to the lumbar spine). This permits us to raise a crucial distinction between referred pain from spine vs limbs: referred pain is uni-directional, and extends in a centrifugal direction (axial-to-distal), not the opposite [46-49]. Table 2 summarizes data from Mellin and Hurri [50] on referred pain distributions from lumbar spine vs hip in samples of low back and hip pain patients.

**Table 2:** **Mellin and Hurri**[50]**: Back vs hip pain referral frequency (%)**

	**LOW BACK**	**HIP**
BACK	–	0
BUTTOCK / GROIN	–	62
THIGH	18	6
LEG	37	2
FOOT	20	0
BUTTOCK + LEG / FOOT	–	6
TOES	26	0

Of note is the complete lack of referral of hip pain to the spine and the much greater incidence of pain in the lower leg and foot arising from spinal pain. Interestingly, there were no subjects reporting groin pain referral from the low back, even though numerous experimental investigations have shown this to be common [51,52]. From this limited database, it seems that hip pain is more localized and has a smaller extent of pain referral than lower lumbar pain and that it refers pain only centrifugally, not proximally into the spinal area.

This situation is somewhat mixed with respect to the mid-limb. Witting et al. [53] injected an algogen into the brachioradialis muscle near the elbow in normal humans and found that, while the majority of pain referral was distally, there was some proximal referral to the shoulder.

This situation is even more clear-cut in the distal limb where experimental pain in the tibialis anterior is confined to a small local spread and then refers to a small, very discrete zone distally in the ankle. There is no spread or referral of pain proximally [54,55].

### Clinical phenomenology: summary

The data reviewed above provide support for the premise that there is a difference in the localization and referral patterns of pain arising from spinal joints vs peripheral joints and muscles. The question is, why this is so?

Why is pain referred more frequently from the spine vs the peripheral tissues?

Why is pain referral from the spine more extensive and more widely distributed?

Why is pain referred uni-directionally from the spine to the limbs and not in the reverse direction?

### Explanation of thesis

In order to provide an answer to the questions asked above, a systematic exploration of the relevant neuroanatomy along with the functional characteristics of these structures will be undertaken. The starting point will be in the extreme periphery, with the neural receptors in the spinal joints.

1. Nociceptors:

i. Types:

Ever since the seminal work of Wyke [56-58] who identified the major types of joint mechanoreceptors, much more has been learned about the innervation of the spinal joints, including their innervation by pain-sensing receptors – nociceptors. Early work by Giles [10-13] identified several important features. By using substance P immunohistochemistry, Giles localized receptors sites in the facet joints, proving that they were pain-sensitive structures. Following that, histological studies demonstrated free nerve endings in the synovial folds and the facet joint capsules. Additional work in this area has been conducted by Cavanaugh [15,16] as well as McLain and Pickar [17].

It is evident that there are no differences between the types of mechanoreceptors (including nociceptors) in spinal joints vs peripheral joints. This mechanism cannot be used to explain any of the features of spinal pain that have been outlined above.

ii. Density:

It is on the matter of density of nociceptors, that there may be an important difference which could contribute to our explanation. This would follow from the axiom noted above that density of mechanoreceptors is strongly correlated to acuity of tactile discrimination. McLain and Pickar [17] showed that the facet joint nociceptor density is rather sparse as compared to appendicular joints. As well, it has been shown that the receptive fields of facet joint nociceptors are comparatively large [59-61]. This latter point is the functional manifestation of the low receptor density.

2. Afferent fibres

i. Types: Following from Wyke’s work described above, there are no differences in the major types of afferent fibres innervating the tissues of the spinal facet joints or the deep spinal somatic structures (A-alpha (Ia), A-beta (I-b, II) A-delta (III) or C (IV) fibres).

ii. Segmental ptorigin: With regard to the lumbar spine, each lumbar facet joint receives innervation from the same segment and from several segments above and below. Recent investigations reveal that the local segmental innervation is supplied via somatic nerves in the posterior primary ramus, while distal segmental innervation is supplied via fibres that run in the posterior sympathetic chain [14,29,30,62-64].

**Figure 6:**
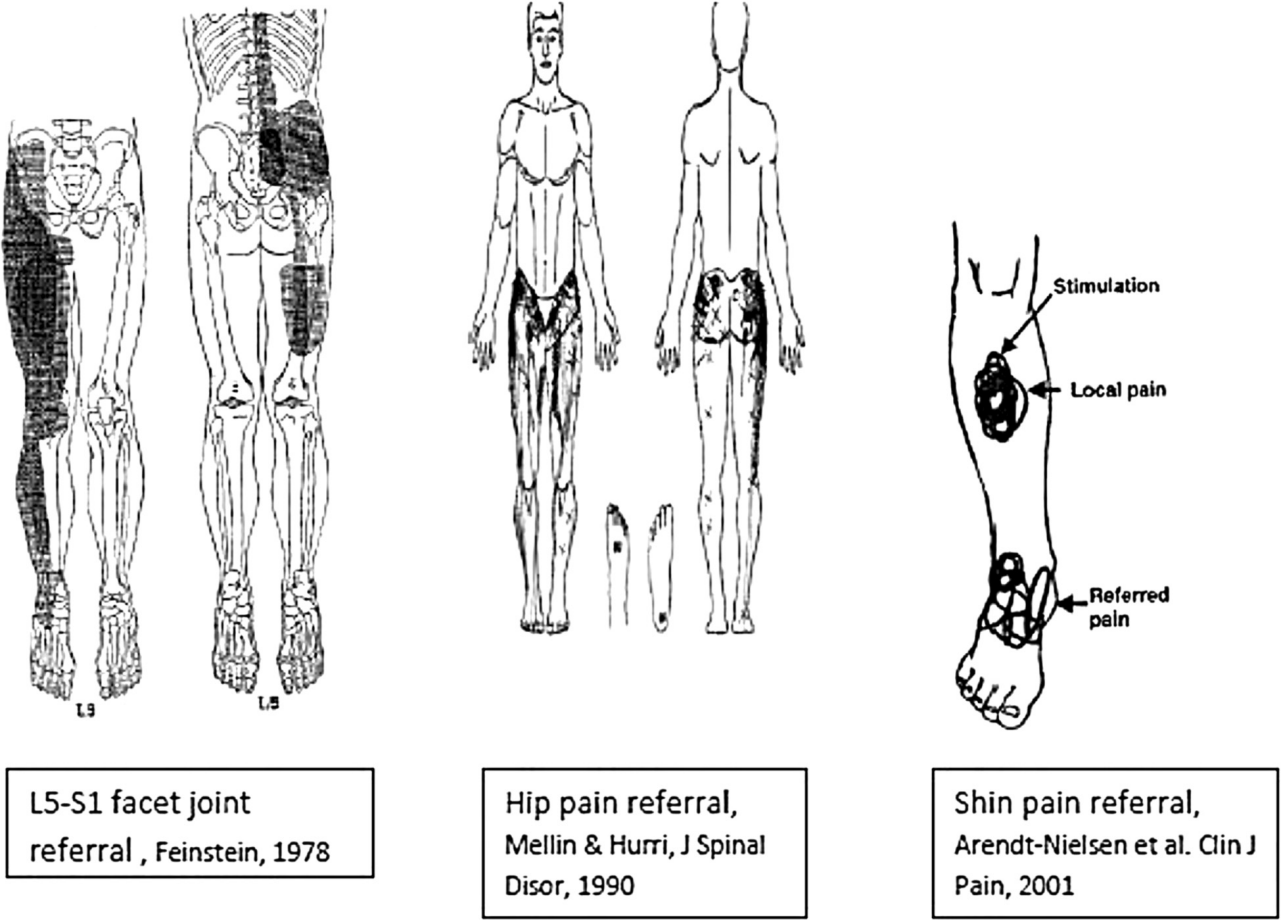
**Origins of innervation of L5-6 rat facet joint [**[63]**] Ishikawa et al. Eur Spine J 2005;14: 559–564.**

**Figure 7:**
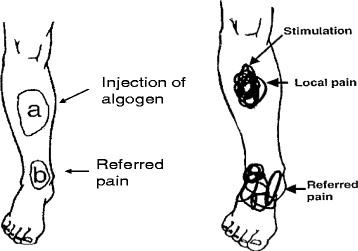
**Origins of innervation of the multifidus muscles at L5-6 in the rat.** Taguchi et al. Neurosci Lett 2007;427: 22–27 [64].

Figures 6 and 7 demonstrate the dorsal root ganglion of origin for innervation of both the facet joints and multifidus muscles, respectively, of the L5-6 spinal level in the rat. Similar findings related to the intervertebral disc have also been reported [65-67]. It is clear that the level supplying the largest proportion of primary afferent fibres to the L5-6 level is the L3 level. These studies have been interpreted to explain the referral of pain from the L5 disc (and facet/ posterior muscles) to the groin area. More importantly, these studies show that the spinal joints have multi-segmental innervation, in some cases, from as many as 5 levels. This diversity of innervation origin is very likely to be a major source of explanation for the poor discriminability and high degree of pain referral from spinal structures.

**Figure 8:**
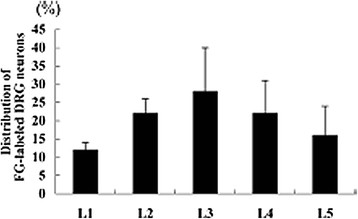
**From Gillette et al., 1993 [**[2]**].**

A similar multi-segmental origin of innervation has been demonstrated for the cervical spine. Ohtori’s group [68-70] found nociceptive afferents which innervated the C1-2 posterior joint arising in dorsal root ganglia (DRG) from C1-C8, from C1-T2 for the C3-4 facet joint and from C3-T3 for the C5-6 facet joint. As with the lumbar spine, afferent fibres from lower cervical and upper thoracic DRG’s run in the posterior sympathetic chain. Fitz-Ritson [71] and others [72-75] have found that cells from the C2 dorsal root ganglion terminate in a wide variety of cranial and caudal structures including: Laminae II, VII and VIII of the spinal cord (to as low as the upper thoracic cord), the hypoglossal, medial vestibular, lateral cuneatus nuclei and lateral parvocellular reticular formation.

iii. Afferent fibre peripheral branching:

Several authors have reported that some primary afferent fibres to spinal tissues branch out to terminate on several divergent structures including the facet joint, the deep spinal muscles as well as more distant tissues in the groin [76-78]. This phenomenon has been suggested as one of the reasons for referral of spinal joint pain to the local muscles as well as to the groin. It is unlikely that this mechanism can explain any of the other more distal pain referral patterns.

iv. Primary afferent arborisation (input divergence/rostro-caudal projection):

Not only does each spinal segment receive afferent terminations from multiple segmental levels, but individual primary afferent fibres make a highly divergent pattern of termination in the spinal cord including extensive rostro-caudal projection, projections to multiple dorsal horn (DH) laminae and projections bilaterally. Figure 8 shows the results Gillette et al.’s studies of a labelling tracer into the right L5-6 facet joint in the rat [2,3]. Gillette et al. [2,3] described that the afferent fibres innervating that joint “terminate bilaterally in laminae I-II, V-VII and X of the lumbar, sacral and thoracic spinal cord”. At that time, it was not clear if this represented divergent arborisation of individual afferent fibres to multiple spinal levels or, as has been shown since, the result of the multi-segmental innervation of the single facet joint (and surrounding deep segmental muscles). Gillette et al. stated the following summary which is a touchstone for the current thesis:

“These diffuse patterns of terminal arborization are reminiscent of visceral input and contrast with the more restricted unilateral terminal fields reported for afferents innervating more distal hindlimb tissue” [2].

In subsequent writing, Gillette also surmised that this distinctive spinal innervation pattern contributed significantly to the qualities of spinal pain as diffuse, poorly localized and frequently referred distally [8]. Furukawa et al. [62] confirmed the bilateral projections from unilateral facet joints in the cat.

In summary, spinal afferents appear to diverge considerably as they terminate in the dorsal horn to include multiple segments of input termination as well as bilateral input termination. These features are in contrast to the pattern observed for afferent fibres from distal or peripheral structures which terminate in fewer segments and, most often, only unilaterally [2,8].

I will now examine the dorsal horn neurons (DHN’s) themselves in regard to features that might distinguish between pain from spinal vs peripheral joint (distal) structures.

3. Dorsal horn neurons: There are four issues pertaining to dorsal horn neurons that are pertinent to our thesis: i. location of dorsal horn cells upon which afferent fibres from spinal vs distal tissues terminate – medial to lateral somatotopy; ii. location of DH cells of termination of afferent fibres from spinal vs distal tissues – laminar organization ; iii. background activity of DHN’s , and, iv. types of dorsal horn neurons: ‘nociceptor specific’ (NS) vs ‘wide dynamic range’ (WDR) neurons as well as the patterns of convergence onto DHN’s which are responsive to inputs from deep spinal tissues.

i. Medial-to-lateral somatotopy:

As early as 1986, Molander and Grant [79] had identified a “highly ordered somatotopic” organization of dorsal horn neurons whereby afferents from the hindlimb preferentially terminate on DH cells located in the medial aspect of the dorsal horn. In 1991, Bullitt [80] confirmed this somatotopic organization of afferent input from structures in the peripheral limb. He reported that afferents from distal tissues (toes) terminated in DH neurons that were represented most medially in the DH. Afferents from the hip (proximal tissues) were more laterally placed. King and Apps [81] confirmed this pattern for the forelimb in the rat, when comparing digital (predominantly medially) and shoulder (more laterally located) afferent terminations (using c-Fos expression) in the cervical dorsal horn.

**Figure 9:**
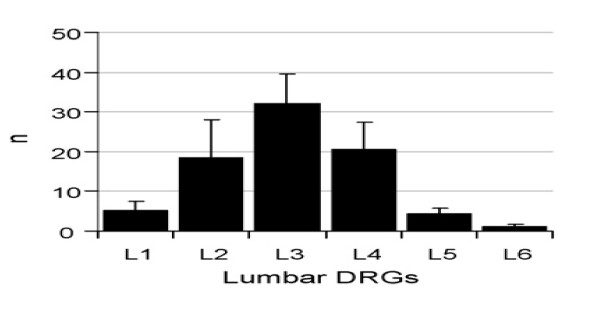
**Gillette et al. [**[8]**]. Left side of figure shows lateral location of “low back neurons”.** Right side of figure is discussed below under “convergence”.

**Figure 10:**
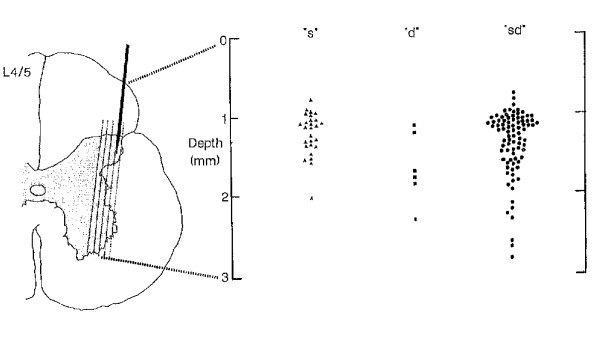
**Ohtori et al., Spine 2000 [**[82]**].** Note the strong filling in the lateral aspect of the dorsal horn.

**Figure 11:**
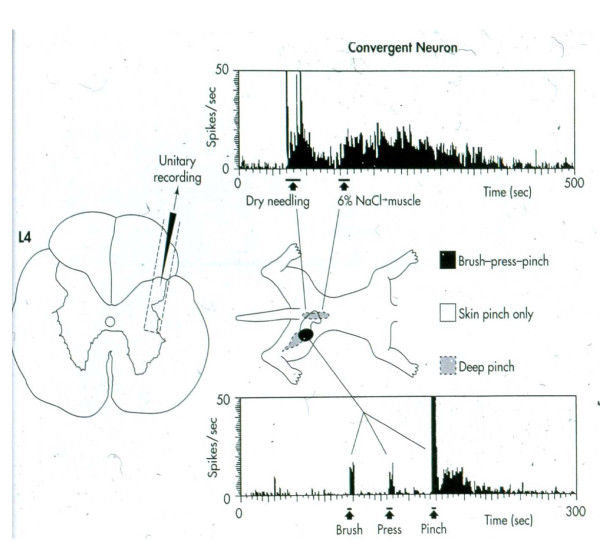
**A, L3 facet injection; B. L5 facet injection.** T. Taguchi et al. / Neuroscience Letters 427 (2007) 22–27 [62]. Note the strong filling in the lateral aspect of the dorsal horn.

**Figure 12:**
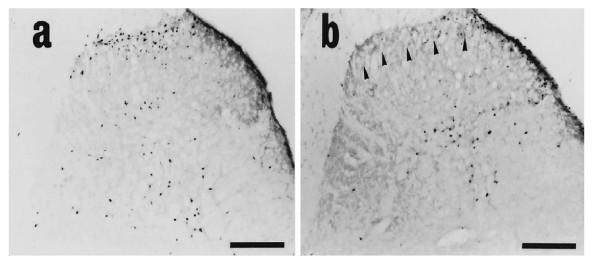
**Schematic representation of the organization of the primary afferent inputs into the dorsal horn showing the somatotopic arrangement of inputs from peripheral, proximal and axial tissues (Hu et al., 2005, [**[83]**].**

Gillette et al. [1-3] appear to be the first to report the identification of dorsal horn neurones onto which afferent fibres from deep spinal tissues terminated, calling these neurons “low back neurones”. Their single unit recording studies identified the location of these neurons as “exclusively in the lateral dorsal horn” [6], [8] (pp. 354)]. Figure 9 replicates this work and demonstrates the receptive field of the neuron under recording. Since that time, considerably more support has emerged for this finding [67,82-84] (See Figures 10, 11 and 12).

With regard to the upper cervical cord, similar findings have been documented by Hu et al. [85]. In their study on cranio-facial inputs to the upper cervical dorsal horn, they found that those inputs related to “peripheral” structures, i.e., those innervated by the V3 division of the trigeminal nerve (peri-oral tissues), terminated in the dorso-medial part of the upper cervical cord, while V1 inputs (more axial) and those from the deep upper cervical paraspinal tissues terminated in the most ventrolateral part of the upper cervical cord. Inputs from V2 were represented in between. In another study, afferent terminations from the superior sagittal sinus [86] were found in the most ventrolateral part of the C1-C3 spinal dorsal horns on both sides. This is reminiscent of a similar mapping of visceral and axial afferents from the thorax/abdomen to the lateral aspect of the thoraco-lumbar dorsal horn noted above.

These and other [87-92] studies provide ample evidence for a somatotopic medial-to lateral organization of afferent inputs that conforms roughly to the following pattern: peripheral / distal inputs are largely located in the medial dorsal horn in the lumbar and cervical regions; axial inputs are primarily located in the lateral dorsal horn and proximal tissue inputs are intermediary. This appears to indicate that, at the first level of integration in the spinal cord, the nervous system processes sensory inputs (at least nociceptive inputs) in two different compartments: axial vs distal or spinal vs limb tissues of origin. Figure 12 demonstrates this schematically for the entire spinal cord (from Hu et al., 2005 [85] and with data from King and Apps, 2000 [81]).

As Gillette et al. [8] noted (above), this pattern is reminiscent of the pattern of input for the viscera whose DH cells of termination are also more often laterally located in the thoraco-lumbar spine.

Petko and Antall [92] have added several important aspects to the medial-lateral somatotopy in the lumbar dorsal horn. First, they confirmed that laterally located cells receive multi-segmental input, from all segments in the lumbar spine, whereas medially located cells receive input from only up to 3 segments. Second, they reported that medially-located cells project to laterally located cells within the same levels, but that the reverse does not occur. Third, they reported that laterally located cells establish commisural connections with the contralateral lateral DH, but the reverse (for the medial DH) does not happen at all. Several of these findings confirm those of Gillette et al. [1-8] from several years earlier.

ii. Dorsal horn somatotopy: laminar organization

**Figure 13:**
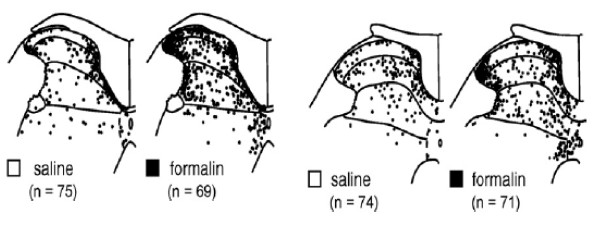
**Taguchi et al. Pain 2008;138:119–129 [**[93]**].**

From the work of Ohtori et al. [84] (Figure 10) above, it can be seen that afferent inputs from deep tissues in the spine do not terminate in Lamina II (as opposed to the inputs from cutaneous sources, which do). Lamina II cells are important in pain processing, as they evoke inhibitory outputs on Lamina I cells. This feature of deep spinal input may contribute to the development of chronicity of spinal pain; however, additional research is needed here to fully explicate the contribution of this feature to the characteristics of deep spinal pain.

iii. Background activity: Taguchi et al. [93] have shown that “low back neurons” have greater levels of background activity than do non-low-back neurons, as seen in Figure 13. The physiologic significance of this feature is not clear, but it may relate to the development of central sensitization by virtue of different characteristics of spatial summation in “low back neurons”.

iv. Dorsal horn cell type and afferent convergence

Gillette et al. were also the first to describe the “low back neurons” as ‘hyperconvergent’, meaning that they received input from a wide variety of tissue sources [1,2 6–8]. The categories of these tissue sources include the ones we have already encountered – the axial / proximal / distal axis – as well as three other important categories: noxious vs non-noxious, superficial (cutaneous) vs deep and somatic vs visceral. Evidence for both the lumbar and cervical spine for each of these categories will be examined in order to support the contention that dorsal horn neurons that receive input from spinal (axial) tissues are “hyperconvergent”. This will be contrasted with DH neurons which subserve sensation from peripheral limb tissues.

DH neurons with exclusively noxious input are known as Nociceptor Specific (NS) cells, while those receiving both noxious and non-noxious inputs are known as Wide Dynamic Range cells (WDR). Gillette et al. [2,3] found a predominance of WDR cells (77 vs 23%) in their sample of “low back neurons”. They used this finding to characterize these neurons as “hyperconvergent”. However, it is not known what the percentage is of NS / WDR cells in the more medially located “peripheral limb” cells, making comparisons between spinal vs limb mechanisms speculative here.

In the upper cervical spine, Hu et al. [94] and Sessle et al. [95] found a similar result. Sources of input to NS and WDR cells in the C1 and C2 dorsal horns are shown in Table 3, and include facial (via trigeminal nerve), tongue, TMJ and perioral structures as well as deep neck inputs.

**Table 3:** **Input to Vc, C1 and C2 neurons. Adapted from Hu et al.**[84]

	**LTM %**			**WDR %**			**NS %**		
	**rVc**	**C1**	**C2**	**rVc**	**C1**	**C2**	**rVc**	**C1**	**C2**
**Neuron numbers**	272	25	47	51	13	18	46	18	25
Spontaneously active	9	8	18	62	38	44	27	11	17
Peripheral afferent inputs:									
Response to noxious heat	0	0	0	90	77	94	81	84	73
C afferent (cutaneous)	1	0	0	80	100	94	50	61	36
XII nerve	4	8	4	51	64	81	24	45	63
Mechanoreceptive field (involving…):									
Only 1 V divisions	90	63	87	13	23	22	46	28	67
2 V divisions	9	37	15	22	50	50	28	55	25
3 V divisions	1	0	0	65	34	28	26	17	8
V, in total	100	100	100	100	100	100	100	100	100
Intraoral component	4	0	0	51	25	44	50	56	38
Perioral component		4	0		9	6		27	0
Neck, occiput, or back of Ear (C2/C3)		0	30		46	72		28	46
Intraoral component only		0	0		0	11		0	0
Perioral component only		4	0		9	0		22	0
Neck, occiput or back of ear (C2/C3) only		0	6		0	0		0	0
Tongue (deep)		0	0		13	33		29	30
TMJ deep component		0	0		40	65		63	58

Most DH cells receive some cutaneous input. Some cells (called ‘superficial neurons’) receive only input from the skin, while a proportion of other DHN’s receives inputs from skin and from deep tissues [96,97]. These neurons have WDR characteristics. Hu et al. [94] found a very small sample of cells with only deep inputs. These characteristics are probably similar for both spinal and non-spinal DH neurons; however, in the case of low back or upper cervical neurons, skin input becomes more important as a contributor to hyperconvergence.

**Figure 14:**
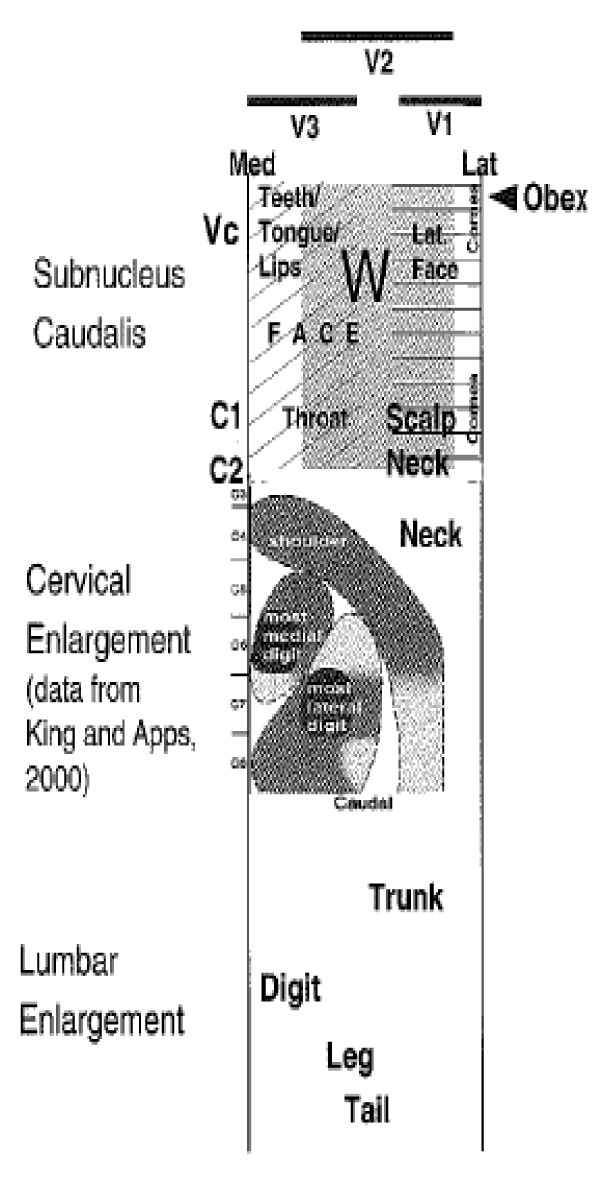
**Receptive fields of a “low back neuron”.** Gillette *et al*. [8].

While non-spinal DH neurons (i.e., those receiving input from the peripheral limbs) do receive deep inputs, they are generally less widely distributed and are more local to an area of injury. Gillette et al. [2,3] found much more widespread sources of deep inputs from paraspinal muscles, ligaments, discs, periosteum, facet joints, dura and skin (of the back) as well as tissue of the hip and leg (as shown in Figure 14).

Gillette et al. [2,3] also found that low back neurons received convergent input from visceral inputs as well as somatic sympathetic inputs (see Suseki et al. [14] and Jinkins [29,30] above for L1-3 input via the sympathetic trunk). This wide range of input sources – superficial, deep somatic, local and distant somatic structures, noxious and non-noxious and, finally, visceral and sympathetically-mediated – prompted the terminology of “hyperconvergence”. Gillette et al. contrasted this pattern of input with the pattern involving “spinal neurons serving the limbs” as “inputs (from) fewer individual tissues over very small areas, often just from the skin” (citing their own work [2,3] as well as Fields and Heinricher [98] and Price [49] as evidence for the peripheral joint DH input patterns).

A similar situation exists with respect to the upper cervical DH neurons which are part of the trigemino-cervical nucleus and which participate in pain processing in the oro-facial-cervical region. Wide ranging sources of inputs have been found in single-unit survey studies [94,95,99-101].

Gillette [1,6,8] and many others [102-105] have indicated that convergence of afferent inputs onto DH cells is a likely candidate mechanism to explain referred pain from deep somatic and visceral sources. These studies are based upon the theory developed by many, especially Ruch [27] known as “convergence-projection”. For example, convergence of visceral inputs onto DH neurons receiving input from spinal deep structures (such as in the thoracic spine [102,103]) has been proposed as the mechanism for referred visceral pain to the back region. Convergence of peripheral limb afferents onto “low back neurons” (as in Gillette et al., above) is thought to explain referral of pain from the low back into the limb. The fact that pain in the peripheral limbs does not refer to the spine (see above) is likely explained by the converse situation – DH neurons subserving those inputs (medially-located cells) do not receive convergent input from any other sources. Here, a critical distinction between “low back” (or “spinal” to include the cervical region) DH and non-back DH neurons comes to light.

v. Convergence and central sensitization

A critical feature of convergence is the link between the multiplicity of inputs and the development of central sensitization. It has been known for many years that the convergence of both superficial cutaneous and deep (somatic or visceral) inputs has the capacity to augment the development of central sensitization [106]. This was first shown for inputs from the lower limb by Wall and Woolf in 1986 [107]. The question arises as to whether “low back” (or “spinal”) DH neurons are particularly susceptible to the development of central sensitization as a result of the wider range of inputs, including those which have come to be called “silent afferents”.There are numerous methods for evaluating the development of central sensitization in the spinal cord as a result of experimental and clinical noxious inputs. We will now discuss two of these, as they have been used to study the behaviour of DH neurons receiving input from spinal tissues.

a. EMG studies

**Figure 15:**
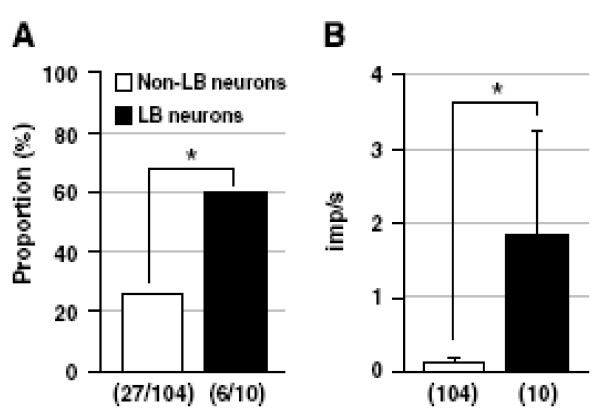
**TMJ injection (Yu et al., [**[109]**]).**

**Figure 16:**
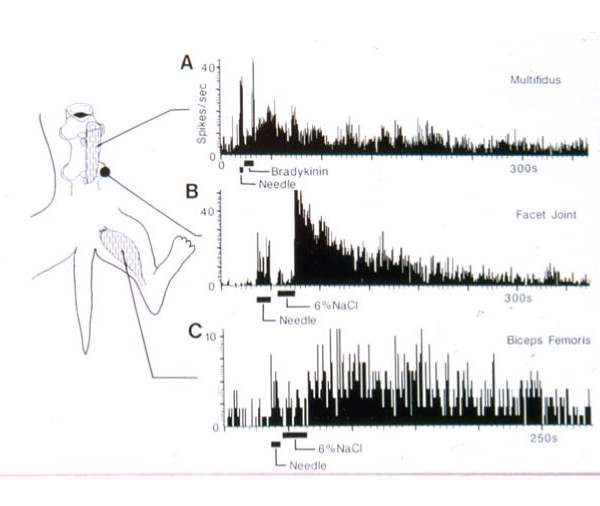
**Neck injection. (Hu et al., [**[108]**]).**

**Figure 17:**
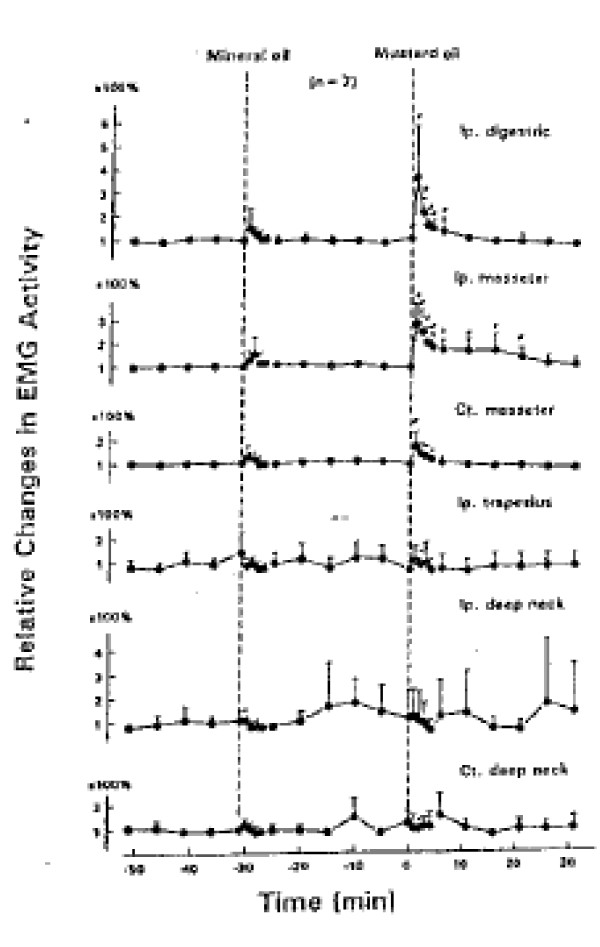
**Sagittal sinus injection. (Hu et al., [**[110]**]).**

Hu et al. conducted a series of studies using EMG recordings of the activity of a variety of cranio-cervical muscles in response to mustard oil-induced irritation of several different tissue sources: deep paraspinal tissues at C1-C2 [108], TMJoint [109], and posterior cranial vasculature and dura [110]. The results of these studies are displayed in Figures 15, 16, 17. My analysis in the present report will consider the TMJoint to be similar to a “peripheral joint”, just as the hip joint would be to the lumbar spine. It can be seen in Figures 15 and 17 that, as a result of deep spinal or vascular irritation, EMG activity is increased in all muscles tested, including those around the posterior cervical (spinal) region as well as those associated with the “peripheral” joint (TMJ); however, the reverse is not the case. TMJ irritation does not evoke increased activity in the neck-related muscles (Figure 16).Hu et al. [94] and Sessle et al. [95,111] have interpreted this to mean that the pattern of evoked muscular activity, which is dependent on sensory-motor processing in the trigemino-cervical nucleus, is more divergent or widespread for spinal and vascular (visceral?) inputs than for peripheral joint inputs. This suggests different sensory-motor processing of noxious inputs for these two different categories of tissue sources and also shows that spinal pain-related processing involves a more divergent pattern of inputs and outputs as compared to the process subserving noxious input from the peripheral joints.

b. Receptive field expansion

The type of DH neurons discussed above (responsive to noxious vs non-noxious input; responsive to skin and or deep inputs; responsive to local vs distal inputs) are typically studied and characterized by experimental methods of applying standardized provocations to tissues under single-unit extracellular recording protocols. This allows the experimenter to determine if a particular tissue (typically skin) is innervated by an afferent fibre which terminates on that DH cell. Essentially, this exercise identifies the DH neuron’s “receptive field”. Non-noxious stimuli typically involve brushing the skin or applying innocuous pressure. Noxious inputs may be cutaneous, consisting of pinching or the application of superficial irritants, or deep, consisting of the application of various algogenic substances, typically by injection or by applying deep pressure.

The results of many such studies allows these neurons to be characterized as to their sensory type - low threshold mechanoreceptor (non-noxious), NS or WDR. As well, it permits the delineation of their receptive fields to each of the modalities in each of the tissues being mapped. When noxious input to DH neurons induces an expansion (in the short term) of one or more receptive fields, a currently widely-held explanation is that this occurs because of the development of central sensitization. In simpler terms, as a result of noxious stimulus, the responsiveness of the neuron undergoes a “shift to the left”, with the threshold for responsiveness lowered and a state of hyper-responsiveness induced.

This experimental model has been applied by two groups to the noxious input from paraspinal / axial tissues in the lumbar spine and the upper cervical spine. Gillette et al. [1,5-8] have reported on the changes of the extent of the receptive fields of DH neurons following algogenic stimulation in a variety of local (spinal) and distal (hindlimb) tissue sites. They report that the extent of RF expansion observed in low back neurons in response to painful stimulation of deep spinal tissues is larger than that observed in non-spinal (“peripheral”) neurons.

Vernon et al. [112] have shown similar effects in the upper cervical cord where mustard oil-induced (painful stimulation) expansion of receptive fields is similarly quite extensive and larger than those observed for studies involving TMJoint irritation.

**Figure 18:**
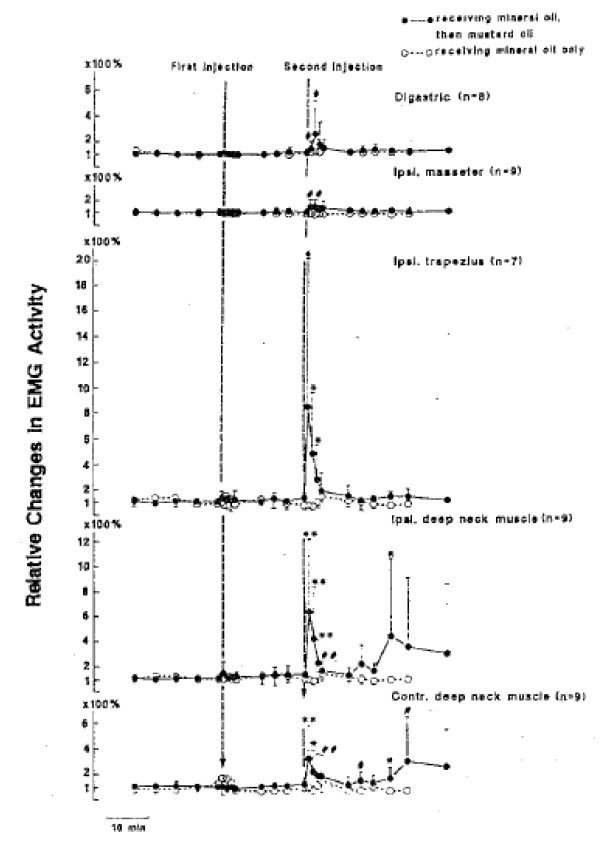
**Location of single neurones recorded in Hu et al. [**[84]**].**

Figure 18 shows the location of C2 dorsal horn neurons sampled in Hu et al. [84] showing that neurons located in the deeper laminae V-VI generally have larger receptive fields. Most of the neurons here that receive input from cervical muscular sites are located in the lateral aspect of the deeper layers.

**Figure 19:**
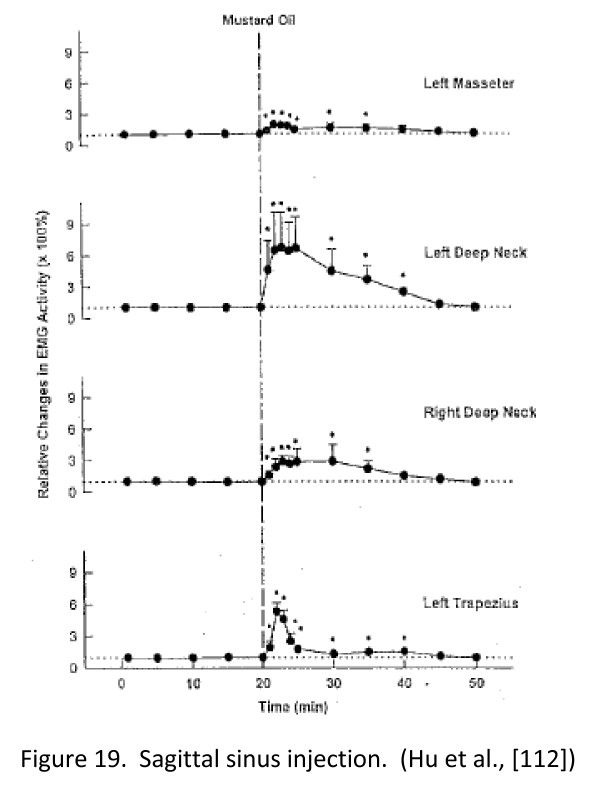
**Expanded receptive field of a C1 neuron following mustard oil injection into the deep C1-2 paraspinal region on the left.**[112].

Figure 19 shows the response of similar neurons to injection of mustard oil into the deep upper cervical paraspinal tissues in terms of changes in the receptive field to non-noxious and noxious stimulation [112]. This expansion is indirect evidence of the effects of central sensitization.

Makowska et al. [113] have also provided support for the wide-ranging motor activation pattern from upper cervical pain by showing that hypertonic saline injection into the semispinalis muscle facilitated the Jaw Opening Reflex for at least one hour. The reflex threshold decreased to 61 % after injection (vs controls).

While the data from Gillette et al., Vernon et al. and Makowska et al. are limited in scope, they appear to support the notion that noxious input from spinal tissues more strongly evokes central sensitization as compared to inputs from peripheral joint tissues on non-spinal (“peripheral”) neurons. This would appear to provide support for the distinctive clinical phenomenology of spinal pain discussed above.

4. Brain

**Figure 20:**
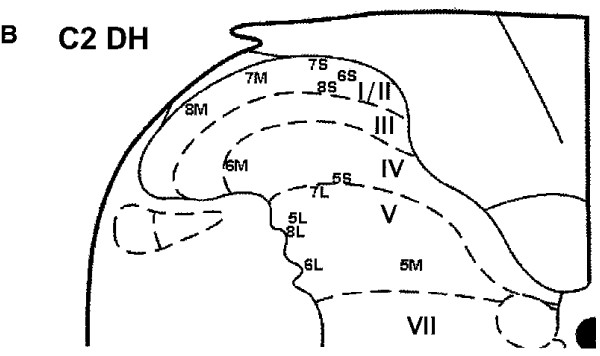
Somatosensory homunculus showing location of spine and torso.

It is evident from classical studies, that the spine (in total) is relatively poorly represented in the somato-sensory cortex (See: Figure 20). The same can be said for studies of changes in the brain resulting from chronic spinal pain. Furthermore, there is virtually nothing known about these changes when comparing deep spine pain vs deep peripheral limb pain. This section will review the few studies that have made tentative steps in these directions and which provide for only tentative conclusions with respect to the thesis of this paper. As well, the much larger and growing area of research into what is called the “pain matrix” in the brain with respect to pain in general will not be reviewed.

It would appear that the first study to explore the projections of low back tissues to the brain was that of Ohtori et al. [83]. They used c-Fos labelling to study the sites in brain that responded to both cutaneous and deep inputs from inflamed spinal tissues and found significant differences, as shown below in Table 4.

**Table 4:** **Brain regions reported by Ohtori et al.,**[83]

**Cutaneous inputs > deep inputs**	**Deep inputs > cutaneous inputs**
• Lateral septal nucleus	• Piriform cortex
• Basomedical nucleus of the amygdale	• Core of Nucleus Accumbens
• Interoanteromedial nucleus of the thalamus	• Basolateral nucleus of the amygdale
• Dorsolateral periaqueductal grey	• CA3 Hippocampus
• Locus Ceruleus	• Ventral tegmental area
• Dorsal Raphe Nucleus	• Ventrolateral periaqueductal grey

Ohtori et al. proposed several conclusions for inflammation-induced pain from deep low back tissues based on these distinctive projection patterns, as follows: 1] The prefrontal cortex and the nucleus Accumbens are part of the dopaminergic system. Activation of these areas would have implications for autonomic function as well as for memory; 2] activation of the ventral PAG could result in quiescence, immobility and hypotension; 3] lack of input into the descending antinociceptive pathways as well as lack of input into Lamina II (see above) may have clinical implications for persistence of pain.

Since this work, Apkarian and colleagues have [114-121] studied brain responses in low back pain patients. It should be noted that, with one exception noted below, these studies involve using patients with chronic low back pain (CBP) as a representative case for chronic pain in general. These findings relate to the present thesis only in so far as they describe brain changes from deep spinal sources, but not in so far as these are compared to peripheral limb sources.

In their 2004 study [117], Apkarian et al. showed that patients with chronic back pain (CBP) showed 5–11 % less neocortical gray matter volume than control subjects. These cortical losses were most evident in the bilateral dorsolateral prefrontal cortex and right thalamus. They commented that this “strongly related to pain characteristics in a pattern distinct for neuropathic and non-neuropathic CBP” [117].

In their 2006 study [120], they found that, in patients with chronic low back pain , those with spontaneous intense pain showed activation of the medial prefrontal cortex (mPFC), including the rostral anterior cingulate region. They described the implication of these findings in the following way: “This mPFC activity was strongly related to intensity of CBP, and the region is known to be involved in negative emotions, response conflict, and detection of unfavorable outcomes, especially in relation to the self” [120]. In a separate group of CBP patients, transient experimental provocation of pain resulted in activation of the insula, which was described as more involved in acute pain reactions.

Their 2008 study is the one most relevant to our thesis. In that study, they compared fMRI patterns of activation in patients with CBP to those with osteoarthritis of the knee [121]. Again, they found that spontaneous CBP had changes predominantly in the mPFC while, in OA patients, mechanical stimulation of the knee resulted in changes more typical of acute pain: bilateral activity in the thalamus, secondary somatosensory, insular, and cingulate cortices, and unilateral activity in the putamen and amygdala.

Taken together, these studies provide support for the proposal that chronic low back pain affects the brain in distinctive ways which may be different from the pattern induced by pain conditions in the peripheral joints (at least as represented by knee joint pain). These results may, at least to some degree, reflect the distinctive features of deep pain from low back tissues that have been described above. Clearly, these findings are very preliminary and only point the way to how much more we need to know about how spinal pain, in particular, affects the brain, and the way in which these changes may underlie the distinctive clinical phenomenology of deep spinal pain.

### Challenges to thesis

One major challenge to my thesis comes from the multi-varied sources of data adduced here in support of the primary thesis, particularly the mix of studies on mechanisms in animals and on clinical phenomenology in humans. It is generally regarded that mechanistic work on small animal models does provide an acceptable analogue to human pain mechanisms, especially at the level of the periphery and the spinal cord [122-124]. However, the degree to which any of the vast amount of work on animal models correlates directly with human clinical phenomena is an ongoing challenge. In the present report, a confluence of evidence from animal and human studies is presented which appear to provide reasonable support for the primary thesis.

A second challenge lies in the circumstances for which a claim of distinction between spinal vs peripheral pain is tenable. In the discussion of referred pain, above, mention was made of various clinical circumstances where our thesis ought not be applied or where such application would be challenging. This situation reflects the complexities inherent in the topic of deep pain as well as the limitations of applying any one theoretical mechanism to that problem and to the problem of chronic pain in general.

The distinction which was already made between somatic referred pain and radicular or neuropathic pain from spinal or peripheral neural entrapment syndromes should not pose a challenge to my thesis as these are clearly distinct pain mechanisms.

Pain behaviors in syndromes which are based on disturbed central pain mechanisms such as in central post-stroke pain and in fibromyalgia pose a modest challenge to my thesis. In these syndromes, normal pain mechanisms at the level of the periphery and spinal cord are substantially altered by centrally-generated mechanisms, making the kind of comparisons between deep spinal and peripheral pain mechanisms which we discuss in this thesis much more problematic [125,126]. Pain referral patterns in these syndromes may not adhere to the strict distinctions we have drawn here between spinal and peripheral limb sources. However, these syndromes are relatively easy to diagnose, so that the clinician is likely to be aware that this is the basis for non-conventional pain referral patterns (especially proximal referral from the limbs, which may be found in some fibromyalgia patients).

The greatest challenge to my thesis appears to arise in regard to the phenomenon of myofascial trigger points (MTrP) either as singular entities or as part of what may be diagnosed as ‘regional myofascial pain syndrome’. Here, we may be required to distinguish between “spread of pain” vs referral of pain. MTrP’s on the torso and in the peripheral limb muscles appear to generate both spread of local pain as well as referral of pain, to distant sites [46,47,127-130]. “Spread” of pain is generally regarded as based on peripheral sensitization [46,47,127,128,130,131] and may be related to local hyperalgesia. As pain or tenderness spread out from the hyperalgesic zone, this may appear to include proximal sites; however, this should be distinguished from proximal pain referral. With regard to actual referral of pain, Simons has opined that at least 85% of MTrP’s refer pain in a distal direction [131], and this is generally in accord with my thesis. In the minority of other cases, perhaps what appears to be ‘proximal referral’ may actually be local spread to include some proximally located distribution within the same muscle or within the same sclerotome. This would make actual referral of pain from a peripherally-located MTrP to the spine itself, much less common whereas the opposite is much more common. This, again, is generally in accord with my thesis.

## Conclusions

In his 2005 review, Gillette summarized the findings of his group with respect to how they contributed to an understanding of the unique features of spinal pain. I have reproduced this Table ( [8], 16–1, pg. 368) and have included additional features which have been reviewed in the current report (Table 5).

**Table 5:** Summary

**Clinical Feature**	**Mechanisms: Gillette, 2005**	**Mechanisms: Current report**
**Poorly localized pain (back, hip and leg; upper neck, TMJ, face)**	Spinal neuron “hyperconvergence” and large receptive fields	Lower density of nociceptors in facet joints, discs (?)
		Afferent branching
**Referred pain from deep tissues**	Nociceptive input to low back neurons from deep tissues more powerful than skin input	Multi-segmental DH input from spinal joints
		Stronger bilateral DH projection from spinal afferents
		Somatotopic organization of spinal vs limb neurons in DH
		Weaker DH medio-lateral interneuronal modulation of pain
		Lack of projection to Lamina II may result in weaker DH inter-laminar modulation of pain
		Hyperconvergence of afferent inputs onto spinal neurons
**Spontaneous, ongoing pain**	After-discharge in many spinal low back neurons after central sensitization (CS) / LTP	Higher levels of ongoing discharge in spinal neurons
**Referred hyperalgesia**	Increased responsiveness (of spinal low back neurons) to mechanical (noxious and non-noxious) input in the receptive field after CS / LTP	Hyperconvergence
**Radiation of pain**	Receptive field expansion following CS / LTP	
	Recruitment of additional low back neurons into activation by:	
	- Release and spread of excitatory substances from afferents	
	- “Unmasking” of latent excitatory synapses by noxious input	
**Persistent referred spinal pain**	Development of CS / LTP produces:	
	- Sympathetically-mediated increases in noxious and non-noxious inputs	
	- Lowered threshold to excitation by non-noxious inputs	
	- Loss of inhibitory controls	

*LTP* = long-term potentiation.

My original goal was to determine if the distinctive clinical phenomenology of spinal pain could be explained by features related to the neuroanatomy and function of the pain system subserving the musculoskeletal tissues of the spine and axial structures. While there has been no definitive experimental test of this thesis, the evidence accumulated in the many studies reviewed here does provide early support for the basic thesis and begins to address the question “What is different about spinal pain?” On the other hand, there are many issues which are poorly explored in the literature. The axial / peripheral paradigm has not been adopted by many clinicians and neuroscientists. This greatly deters the work that is required to explore and explicate my thesis. Myofascial pain may present a particularly strong challenge to the thesis, and much more work on comparing spinal vs peripheral trigger points is needed. In regard to all these issues, this thesis is both speculative and provocative at the same time.

## Competing interests

The author declares that there are no financial or non-financial conflicts of interest with respect to this manuscript. No fees were obtained for the work on this report.

## Author’s contribution

Dr. Vernon is the sole author of this manuscript.
